# Floral Traits and Breeding Systems in *Sincoraea* (Bromeliaceae), an Endemic Genus of Brazilian Rupestrian Grasslands

**DOI:** 10.3390/plants15142184

**Published:** 2026-07-16

**Authors:** Adelly Cardoso de Araujo Fagundes, Jamerson Souza da Costa, Alexsandro Bezerra-Silva, Maria Thereza Dantas Gomes, Mônica Lanzoni Rossi, Isabel Cristina Sobreira Machado, Everton Hilo de Souza, Ligia Silveira Funch, José Alves de Siqueira Filho

**Affiliations:** 1 Programa de Pós-Graduação em Botânica, Departamento de Ciências Biológicas, Universidade Estadual de Feira de Santana, Feira de Santana 44036-900, Brazil; jamersonjsc@yahoo.com.br (J.S.d.C.); sandroufpecav@gmail.com (A.B.-S.); therezadantas18@gmail.com (M.T.D.G.); jose.siqueira@univasf.edu.br (J.A.d.S.F.); 2 Centro de Energia Nuclear na Agricultura, Universidade de São Paulo, São Paulo 13416-000, Brazil; monicalr@cena.usp.br; 3 Programa de Pós-Graduação em Biologia Vegetal, Departamento de Botânica, Universidade Federal de Pernambuco, Recife 50670-901, Brazil; icsmachado@yahoo.com; 4 Fundação Getúlio Vargas (FGV Conhecimento), Rio de Janeiro 22231-010, Brazil; hilosouza@gmail.com; 5 Centro de Referência para a Recuperação de Áreas Degradadas (CRAD), Campus Ciências Agrárias, Universidade Federal do Vale do São Francisco, Petrolina 56300-000, Brazil

**Keywords:** bromeliads, endemic species, pollination, reproductive biology, tropical grasslands

## Abstract

Brazilian *campos rupestres* are a global center of plant endemism, but they are increasingly threatened by climate change and human activities. In this context, *Sincoraea*, an endemic genus of the Espinhaço Mountain Range, provides an important model for understanding how reproductive traits influence species persistence. This study investigated the floral biology, reproductive systems, nectar dynamics, and floral visitors’ interactions of several *Sincoraea* species through fieldwork conducted from 2023 to 2025. Floral biology and pollination were analyzed in four species, while pollen and stigma traits were examined in eight species using light and scanning electron microscopy. The flowers had white tubular corollas, diurnal anthesis, and high pollen viability during anthesis. Nectar production peaked in the morning. All recorded pollinators were hummingbirds and all species were self-incompatible, except *S. burle-marxii*, which was partially self-compatible. The reproductive effectiveness index was 1.00 for all species, suggesting that natural pollination provided effective conspecific pollen transfer without evidence of pollen limitation. These findings highlight the strong dependence of *Sincoraea* on floral visitors and emphasize the importance of conserving plant–floral visitor networks in *campos rupestres*. These interactions are essential for maintaining genetic diversity and the long-term stability of ecosystems.

## 1. Introduction

The Brazilian *campos rupestres* (rupestrian grasslands), a montane system (>800 m a.s.l.) characterized by rocky outcrops and nutrient-poor soils, occupies less than 1% of the national territory but supports approximately 15% of the country’s endemic flora [1,2]. This remarkable diversity is driven by lineage diversification and high levels of species and genus richness, which are distributed across a wide range of habitats shaped by variations in altitude, topography and edaphic conditions. As a result, this predominantly herbaceous and shrubby vegetation is unique on a global scale [3].

Grassland ecosystems are increasingly threatened by anthropogenic pressures, including overgrazing, livestock grazing, agriculture, land speculation, mining, fires, and habitat destruction, a situation further aggravated by the persistent neglect of these ecosystems in Brazil [4,5,6,7,8]. For example, Bromeliaceae is one of the most important, diverse, and conspicuous components of *campos rupestres*, comprising approximately 200 species and 22 genera [9]. However, many of these species are threatened with extinction owing to loss of habitat suitability driven by increasing temperatures, reduced precipitation, and declining fog at mountaintops [1,6,10,11,12].

Among these rare and endangered bromeliads, *Sincoraea* Ule is an endemic genus to the *campos rupestres* of the Espinhaço Mountain Range in Brazil [9]. Ten species occur in the Chapada Diamantina in central Bahia, whereas one species is found in the central portion of the Espinhaço range in northern Minas Gerais. The genus is part of the “Cryptanthoid complex” an endemic lineage from eastern Brazil resulting from the adaptive radiation characteristic of the family [13]. Species of *Sincoraea* do not coexist within the same area, suggesting that they contribute to the expansion of interaction networks in *campos rupestres*, particularly during flowering through the provision of floral resources and, subsequently, during fruiting [14,15,16].

Reproductive studies in plants encompass phenology, floral biology, pollination ecology, and breeding systems, all of which contribute to understand plant adaptations, evolution, and diversification [17]. Such studies are essential for assessing species’ survival capacity and guiding effective conservation strategies [17,18,19], as they enable the diagnosis of reproductive bottlenecks, reveal the structural roles of species within pollination networks, and identify those most vulnerable to habitat fragmentation and climate change [20,21,22].

This is particularly relevant in early diverging lineages, such as *Sincoraea*, which are often characterized by higher levels of self-incompatibility [20,23,24,25], providing insights into how species coexist, interact, and evolve within ecosystems, while also revealing a strong dependence on biotic vectors to ensure reproductive success. Thus, investigating reproductive aspects can support the development of more effective strategies to safeguard species as well as the ecosystems they inhabit [15,26,27,28,29,30,31].

Despite this, comprehensive data on the reproductive biology of bromeliads in Brazil remain scarce, particularly in grassland environments [32]. As an endemic lineage of the *campos rupestres* flora, information about the pollination, breeding systems, and reproductive success can fill knowledge gaps relevant to conservation and management strategies. In this study, we investigated the breeding systems, floral biology, and floral visitors’ assemblages of *Sincoraea* species to characterize their reproductive ecology and evaluate patterns of plant–floral visitors’ interactions in *campos rupestres*. Specifically, we addressed the following questions: (i) What are the floral morphological and biological characteristics of *Sincoraea* species, including pollen and stigma traits? (ii) What are the characteristics of the nectar of *Sincoraea* species? (iii) Which species are its floral visitors? (iv) What breeding systems characterize the species, and how does fruit set vary among the reproductive treatments?

## 2. Results

### 2.1. Floral Biology

The flowers of *Sincoraea* species are white and tubular (see Table 1 for floral dimensions), and are arranged in sessile inflorescences positioned centrally within the rosette. Floral tissues were smooth, and no perceptible floral odor was detected during the field observations. Corolla opening at the apex causes the petals to reflex, resulting in a narrow floral tube across all species. Nectar is the primary floral resource available to floral visitors. Pollen deposition on the beaks of hummingbirds was observed during floral visits. Floral opening is apical, and sepals are smaller than petals; both structures are free. Anthesis is diurnal, initiating between 04:30 and 05:00, and is marked by apical petal reflection and the onset of resources availability. Flowers remained open throughout the day, with senescence beginning at approximately 14:00 and concluding by 17:00, characterized by floral closure and petal torsion. Sunrise in the study region occurs at approximately 05:00–05:30 and sunset at approximately 17:30–18:00, indicating that anthesis occurs at or just before sunrise and senescence is completed well before sunset. This anthesis pattern was consistent across all the examined species.

### 2.2. Stigma Morphology and Receptivity

**Figure 1:**
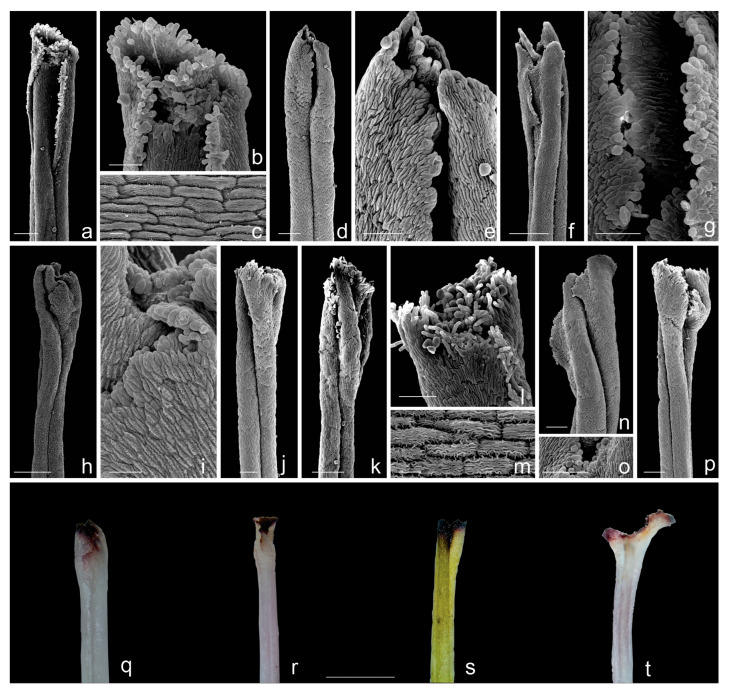
Stigmas of *Sincoraea* observed under scanning electron microscopy (**a**–**p**) and stereomicroscope (**q**–**t**). (**a**–**p**) Stigmatic morphology; (**q**–**t**) stigma receptivity assessed using α-naphthyl acetate + Fast Blue B salt. (**a**–**c**) *S. albopicta*; (**d**,**e**,**q**) *S. amoena*; (**f**,**g**,**r**) *S. burle-marxii*; (**h**,**i**,**s**) *S. hatschbachii*; (**j**) *S. heleniceae*; (**k**–**m**) *S. mucugensis*; (**n**,**o**,**t**) *S. ophiuroides*; (**p**) *S. ulei*. (**b**,**e**,**g**,**i**,**l**,**o**) detail of papillae; (**c**,**m**) detail of the stylar surface. Images are representative of observations from three flowers collected from different individuals of each species. Scale bars: 500 μm (**a**,**d**,**f**,**h**,**j**,**k**,**n**,**p**), 100 μm (**b**,**e**,**g**,**i**,**l**,**o**), 10 μm (**c**,**m**), and 2.5 mm (**q**–**t**).

**Figure 2:**
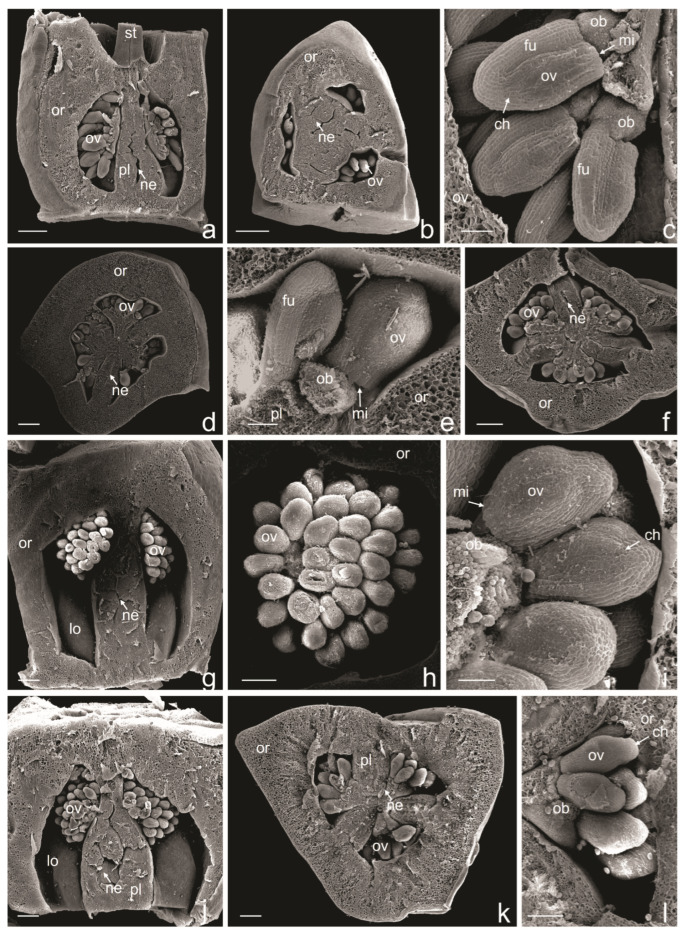
Ovary and ovules of *Sincoraea* observed under scanning electron microscopy. (**a**–**c**) *S. amoena*; (**d**,**e**) *S. burle-marxii*; (**f**–**i**) *S. hatschbachii*; (**j**–**l**) *S. ophiuroides*. (**a**,**g**,**j**) Longitudinal sections of the ovary; (**b**,**d**,**f**,**k**) and transverse sections of the ovary; (**c**,**e**,**h**,**i**,**l**) Images are representative of observations from three flowers collected from different individuals of each species. Abbreviations: ch = chalaza; fu = funiculus; lo = locule; mi = micropyle; ne = nectary; ob = obturator; or = ovary; ov = ovule; pl = placenta; st = style. Scale bars: 500 μm (**a**,**b**,**d**,**f**,**g**,**j**,**k**), 200 μm (**h**), and 100 μm (**c**,**e**,**i**,**l**).

Stigma morphology (simple-erect type) and style morphometry varied among the species (Supplementary Material Table S1; Figure 1a–p). Stigma receptivity assays indicated strong positive reactions (+++) during anthesis in all species tested. Ovule and ovary morphology were consistent among species, with only slight variations in size (Supplementary Material Table S3; Figure 2).

### 2.3. Pollen Morphology and Viability

**Figure 3:**
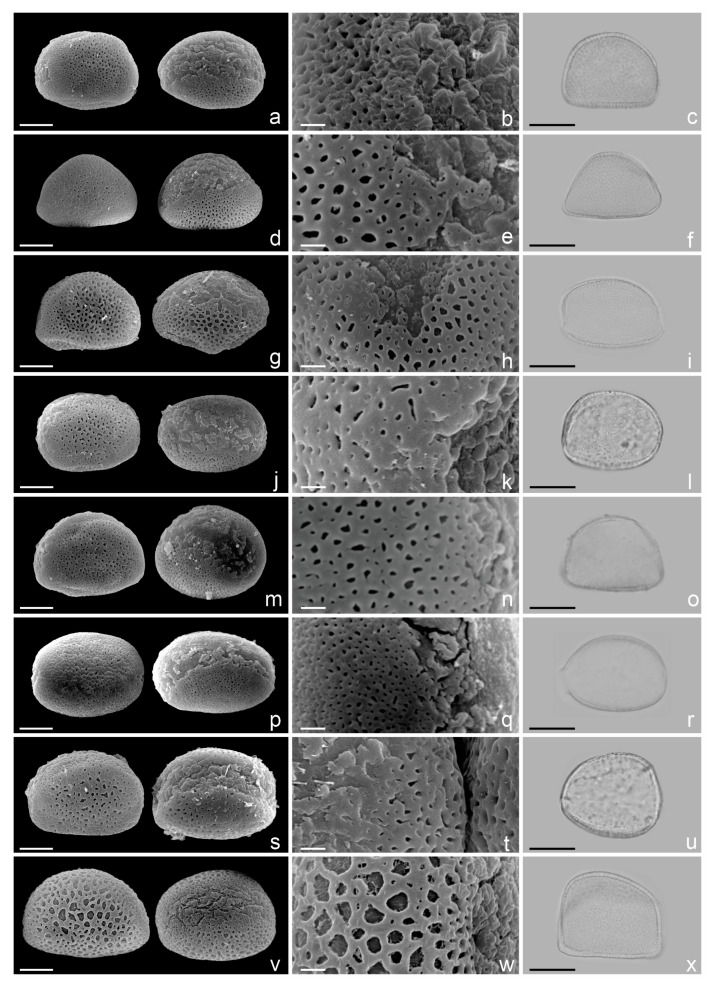
Pollen grain morphology of the genus *Sincoraea*. (**a**–**c**) *S. albopicta*; (**d**–**f**) *S. amoena*; (**g**–**i**) *S. burle-marxii*; (**j**–**l**) *S. hatschbachii*; (**m**–**o**) *S. heleniceae*; (**p**–**r**) *S. mucugensis*; (**s**–**u**) *S. ophiuroides*; (**v**–**x**) *S. ulei*. (**a**,**b**,**d**,**e**,**g**,**h**,**j**,**k**,**m**,**n**,**p**,**q**,**s**,**t**,**v**,**w**) Pollen grains observed under scanning electron microscopy; (**b**,**e**,**h**,**k**,**n**,**q**,**t**,**w**) detail of the aperture; (**c**,**f**,**i**,**l**,**o**,**r**,**u**,**x**) pollen grains after lactic acetolysis. (**a**,**c**,**d**,**f**,**g**,**i**,**j**,**l**,**m**,**o**,**p**,**r**,**s**,**u**,**v**,**x**) 10 µm; (**b**,**e**,**h**,**k**,**n**,**q**,**t**,**w**) 5 µm. Morphometric analyses were based on 25 pollen grains per species. Scale bars: 10 μm (**a**,**c**,**d**,**f**,**g**,**i**,**j**,**l**,**m**,**o**,**p**,**r**,**s**,**u**,**v**,**x**) and 5 μm (**b**,**e**,**h**,**k**,**n**,**q**,**t**,**w**).

**Figure 4:**
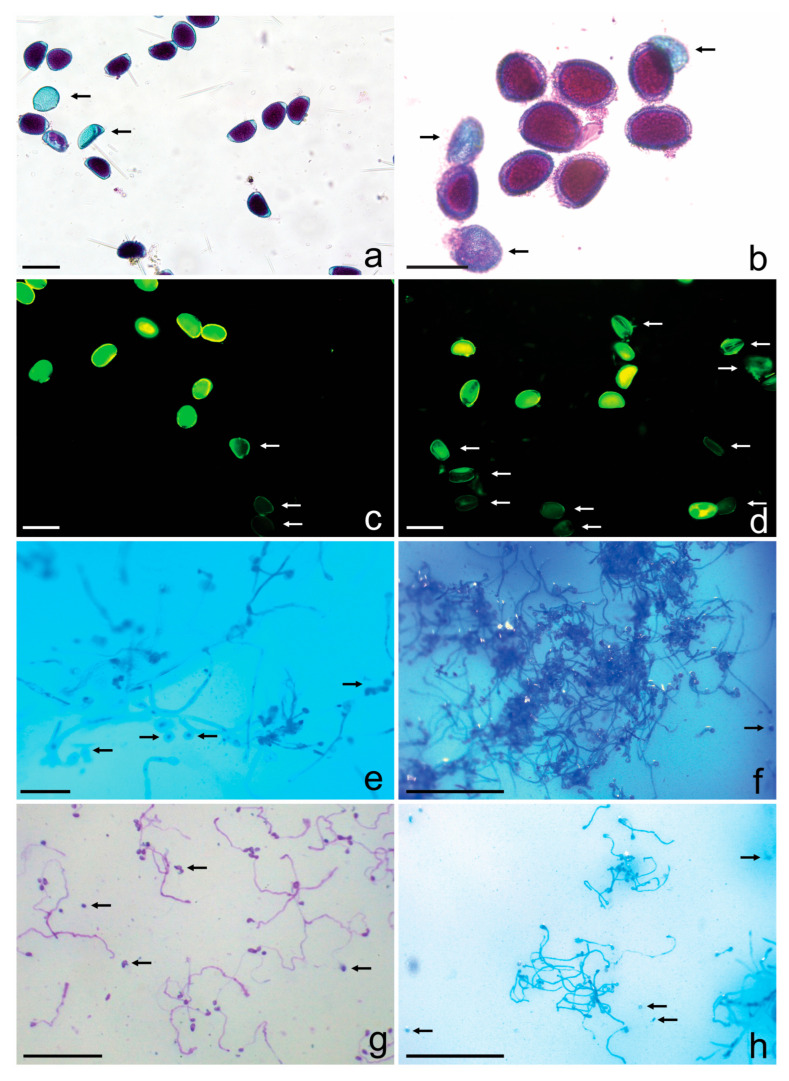
Pollen viability and in vitro pollen germination of *Sincoraea* species during anthesis. Floral species: (**a**) *S. albopicta*; (**b**) *S. burle-marxii*; (**c**) *S. heleniceae*; (**d**) *S. mucugensis*; (**e**) *S. amoena*; (**f**) *S. ophiuroides*; (**g**) *S. ulei*; and (**h**) *S. hatschbachii*. Histochemical tests showing viable and non-viable pollen grains (arrows): (**a**,**b**) 2% Alexander’s solution; (**c**,**d**) fluorescein diacetate. In vitro pollen germination in BM medium (**e**,**f**) and SM medium (**g**,**h**). Arrows indicate non-viable pollen grains. Pollen viability was assessed using three replicates, with 100 pollen grains counted per replicate (n = 300 pollen grains per test). In vitro germination assays were performed with 12 replicates. Scale bars: 500 μm (**f**–**h**), 200 μm (**a**,**c**–**e**), and 50 μm (**b**).

Pollen grain diameter varied among species in both polar and equatorial views, as did exine thickness. All species exhibited oblate, heteropolar pollen grains with circular amb and bilateral symmetry (Supplementary Material Table S4). Pollen grains were consistently sulcate and semitectate. Exine ornamentation was microreticulate in *S. albopicta*, *S. heleniceae*, and *S. mucugensis* and reticulate in the remaining species (Supplementary Material Table S5; Figure 3). Pollen viability during anthesis was high in both Alexander and fluorescein diacetate assays, ranging from 80.25 ± 4.03% to 93.08 ± 3.82%, with no substantial differences between the methods. In vitro pollen germination varied significantly among culture media (*p* < 0.01). Germination in BM medium ranged from 60.83 ± 4.93% to 91.67 ± 3.28%, whereas significantly lower values were recorded in SM medium (5.92 ± 3.53–51.58 ± 4.70%). Pollen tube length followed a similar pattern, with higher values in BM medium (up to 1.67 ± 0.28 mm) and lower values in SM medium (maximum 0.49 ± 0.10 mm; Supplementary Material Table S6; Figure 4).

The ovule number ranged from 72 to 116 per flower, whereas the pollen production ranged from 36,944 to 77,889 grains. Consequently, the pollen–ovule ratio varied from 472 in *S. ulei* to 727 in *S. hatschbachii* (Supplementary Material Table S7).

### 2.4. Nectar Volume and Concentration

**Figure 5:**
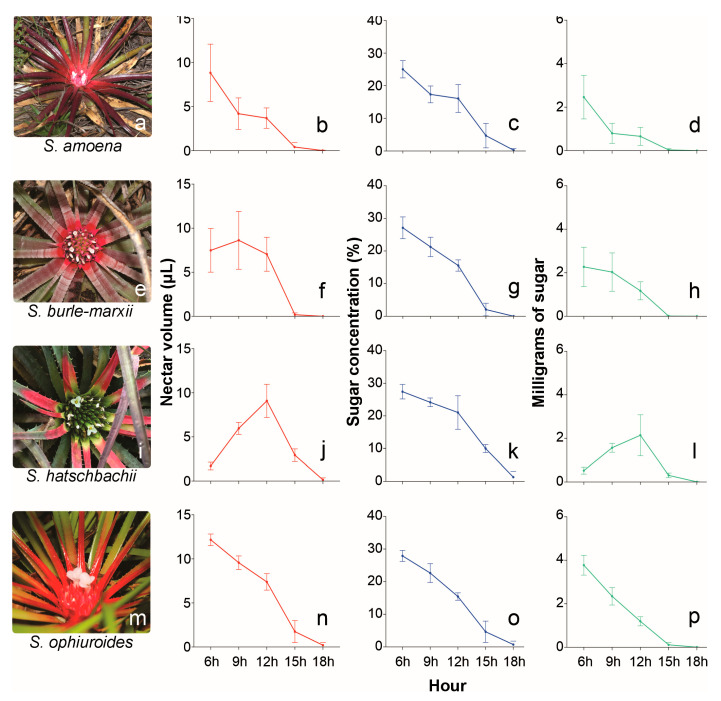
Nectar traits of *Sincoraea*. (**a**–**d**) *S. amoena*: (**b**) nectar volume (µL), (**c**) sugar concentration (%), (**d**) sugar content (mg); (**e**–**h**) *S. burle-marxii*: (**f**) nectar volume (µL), (**g**) sugar concentration (%), (**h**) sugar content (mg); (**i**–**l**) *S. hatschbachii*: (**j**) nectar volume (µL), (**k**) sugar concentration (%), (**l**) sugar content (mg); (**m**–**p**) *S. ophiuroides*: (**n**) nectar volume (µL), (**o**) sugar concentration (%), (**p**) sugar content (mg). Values are based on nectar collected from five flowers of three individuals per species at each sampling time (n = 15 flowers per species per time).

Nectar traits varied significantly throughout the day. Nectar volume, sugar concentration, and sugar content per flower differed among sampling times. Nectar volume remained similar between 06:00 and 12:00, but differed significantly between morning and afternoon periods (*p* < 0.001), with no difference between 15:00 and 18:00. Sugar concentration increased from 06:00 to 12:00 (*p* = 0.004) and differed significantly between most morning and afternoon comparisons (*p* < 0.05), although no differences were detected between 09:00 and 12:00 or between 15:00 and 18:00 (both *p* > 0.05). Similarly, sugar content did not differ among morning measurements (06:00–12:00; *p* > 0.05), but was significantly different between morning and afternoon periods (*p* < 0.001), with no difference between 15:00 and 18:00 (Figure 5).

### 2.5. Pollination

**Figure 6:**
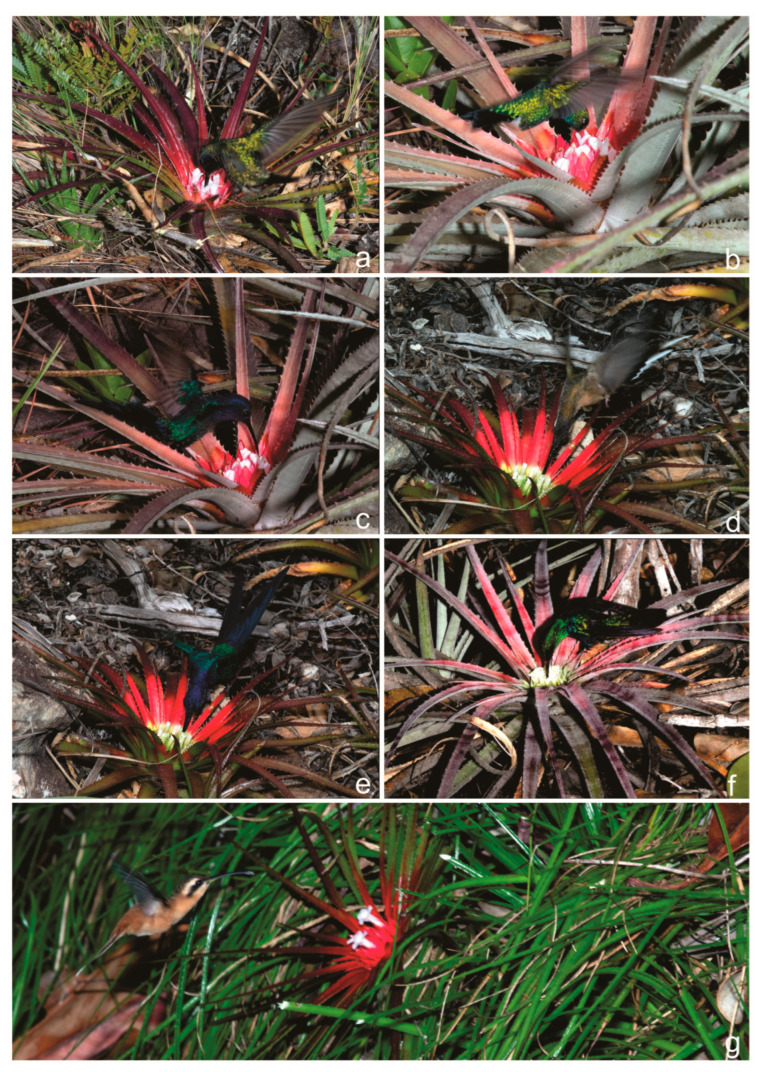
Floral visitors of *Sincoraea*. (**a**) *S. amoena* visited by *Augastes lumachella*; (**b**) *S. burle-marxii* visited by *Chlorostilbon lucidus*; (**c**) *S. burle-marxii* visited by *Eupetomena macroura*; (**d**) *S. hatschbachii* visited by *Anopetia gounellei*; (**e**) *S. hatschbachii* visited by *E. macroura*; (**f**) *S. hatschbachii* visited by *Thalurania furcata*; (**g**) *S. ophiuroides* visited by *Phaethornis ruber*.

During the study period, a single pollinator species was recorded for *S. amoena*, the hummingbird *Augastes lumachella* (Lesson, 1838; frequency = 2.31 ± 0.80 visits h^−1^; Figure 6a). In *S. burle-marxii*, only the hummingbirds *Chlorostilbon lucidus* (Shaw, 1812), *Eupetomena macroura* (Gmelin, 1788), and *Anopetia gounellei* (Boucard, 1891) were observed (overall frequency = 1.90 ± 0.80 visits h^−1^; Figure 6b,c). The pollinators of *S. hatschbachii* included the hummingbirds *E. macroura*, *A. gounellei*, and *Thalurania furcata* (Gmelin, 1788; overall frequency = 2.79 ± 1.74 visits h^−1^; Figure 6d–f). The only recorded pollinator of *S. ophiuroides* was the hummingbird *Phaethornis ruber* (Linnaeus, 1758; frequency = 1.87 ± 0.57 visits h^−1^; Figure 6g).

### 2.6. Reproductive Biology

*Sincoraea amoena* (AI = 0.10; ISI = 0.15), *S. hatschbachii* (AI = 0.10; ISI = 0.11), and *S. ophiuroides* (AI = 0.10; ISI = 0.25) are self-incompatible, whereas *S. burle-marxii* (AI = 0.40; ISI = 0.30) is partially self-compatible (Table 2). In *S. amoena*, treatments differed significantly (χ^2^ = 66.75, df = 4, *p* < 0.001), with higher fruit set under cross- and natural pollination than under spontaneous self-pollination, manual self-pollination, and agamospermy. No differences were detected between cross and natural pollination or among the remaining treatments. A similar pattern was observed for *S. hatschbachii* (χ^2^ = 45.61, df = 4, *p* < 0.001), *S. burle-marxii* (χ^2^ = 55.87, df = 4, *p* < 0.001), and *S. ophiuroides* (χ^2^ = 61.01, df = 4, *p* < 0.001). In all plant species, cross- and natural pollination yielded significantly higher fruit set than spontaneous self-pollination, manual self-pollination, and agamospermy (*p* < 0.001), with no significant differences between cross- and natural pollination or among the remaining treatments. No species produced fruits via agamospermy.

The reproductive effectiveness (RE) index was 1.00 for all species, indicating that fruit and seed set under natural pollination conditions was equivalent to that obtained under supplemental pollination treatments, which indicates the lack of pollen limitation in all *Sincoraea* species.

## 3. Discussion

Based on the reproductive biology data obtained for four *Sincoraea* species (*S. amoena*, *S. burle-marxii*, *S. hatschbachii*, and *S. ophiuroides*), together with morpho-functional analyses of eight species, our results support the hypothesis that the species studied are highly (but not totally) dependent on floral visitors for reproductive success. In these species, the suite of floral traits appear to be associated with the observed floral visitor assemblages in agreement with the pollination syndrome concept. Likewise, nectar traits were associated with visitation patterns and may contribute to reproductive efficiency under natural conditions.

Although flower and beak sizes differed among the studied species, suggesting that morphological matching is not the primary factor structuring plant–floral visitor interactions among the *Sincoraea* species evaluated in this study. In Bromeliaceae, hummingbird pollination systems are frequently associated with floral traits such as tubular corollas, nectar availability, and anthesis timing, rather than a strict correspondence between corolla length and beak size [33,34,35]. Although hummingbirds often act as generalized floral visitors, they may temporarily specialize in a single plant species during foraging bouts, promoting conspecific pollen transfer. Consistent with this, the absence of pollen limitation in all the studied species suggests that pollen transfer was predominantly conspecific [36].

Flowers remained functional throughout anthesis, a common feature in Bromeliaceae [23,24,32,37,38]. Stigma receptivity is linked to floral developmental stages to maximize fertilization success during anthesis [39,40,41]. Most *Sincoraea* species exhibited strong (+++) stigma receptivity during anthesis. Although stigma lifespan is regulated by hormonal control, cell wall modifications, and programmed cell death [42], the ecological significance of this variation among species is unclear.

Species of *Sincoraea*, as well as members of *Cryptanthus* Otto & A. Dietr. and *Orthophytum* Beer belong to the “Cryptanthoid complex,” which is characterized by simple-erect stigma morphology [23,41,43]. This plesiomorphic condition has been associated with increased pollen retention and maintenance of a humid microenvironment on the stigmatic surface, thereby enhancing reproductive success in high solar-exposure environments, such as rock outcrops where many species of the complex occur [44].

Pollen viability was high during the anthesis. In Bromeliaceae, there appears to be a correlation between pollen viability and pollen grain availability, which tends to promote pollen flow and enhance pollination efficiency, particularly in rupicolous environments and in systems associated with hummingbird pollination [31,45,46]. The observed pollen grains were monads, medium-sized, monoaperturate, heteropolar, circular, and had semitectate exine with reticulate or microreticulate patterns. This pollen architecture has been associated with protection against environmental stress in rocky outcrops, which are the typical habitats of the studied *Sincoraea* species [45,46,47].

Another important floral trait contributing to the selection of hummingbirds as primary visitors is nectar characteristics. The data obtained during our study indicate that this floral resource is consistent with typical ornithophilous syndromes [48,49], with volumes ranging from approximately 10 to 30 µL and sugar concentrations of approximately 15–25%. Floral visitors visitation peaks in the morning, coinciding with the period of highest nectar production, which corroborates previous reports indicating that floral visitors activity is directly linked to the timing of nectar secretion [50,51,52]. This temporal matching is crucial for meeting the energetic demands of floral visitors, particularly vertebrates such as hummingbirds, which pollinate while actively flying and therefore expend substantial amounts of energy during foraging [34,50,53,54].

Differences in floral visitor assemblages among the studied species may be associated with variations in flower length, as species with longer flowers tended to receive visits from a greater number of floral visitor species [55]. This pattern is consistent with broader trends in Bromeliaceae, where floral traits play a key role in structuring plant–pollinator interactions, particularly because of the predominance of vertebrate floral visitors, especially hummingbirds [56] and bats [54].

Many bromeliads exhibit strong associations with hummingbirds. Across the family, flowers may be visited by species with distinct morphological traits, such as differences in bill length and width, yet these interactions often form specialized pollination networks [57].

White flowers are not typically associated with ornithophily. However, their contrast with the reddish leaves, bracts, or sepals observed in the studied species appear to provide a sufficient visual signal to attract hummingbirds. Notably, hummingbirds were the only floral visitors observed across the four species evaluated during the study period (2023–2025). Furthermore, the combination of elongated flowers with tubular corollas and conspicuous bracts may favor hummingbird visitation, which is consistent with the exclusive hummingbird visitation observed in this study [58,59].

Predominant self-incompatibility is consistent with patterns reported for many bromeliads and reinforces the importance of floral visitor-mediated cross-pollination in the species we studied. Self-incompatibility is generally maintained because it promotes outcrossing, reduces inbreeding depression, and helps preserve genetic diversity within the populations [60]. In contrast, self-compatibility may evolve under conditions of limited pollinator availability, low mate availability, or demographic instability, where reproductive assurance is advantageous [61]. The high reproductive efficiency observed under natural conditions suggests that floral visitor activity is sufficient to ensure effective pollen transfer. Similar patterns have been reported in other hummingbird-pollinated communities of *campos rupestres*, where floral visitor availability appears adequate to prevent strong pollen limitation [62,63,64,65].

Finally, the indices obtained for the four species evaluated revealed a pattern of predominantly allogamous breeding systems and strong dependence on floral visitors. Although this pattern is consistent with that reported for many ornithophilous Bromeliaceae [20], additional studies are needed before it can be considered representative of *Sincoraea* as a whole. These findings indicate that plant–floral visitor interactions are closely associated with the reproductive biology of the species studied and provide hypotheses that can be tested in additional *Sincoraea* species in the future [66,67].

## 4. Materials and Methods

### 4.1. Study Area and Species

Field observations of floral biology and pollination were conducted in three municipalities between 2023 and 2025. *Sincoraea amoena* was studied in the Municipal Natural Park of Pai Inácio (12°27′27″ S, 41°28′23″ W), in Palmeiras; *S. burle-marxii* and *S. ophiuroides* were investigated in the Municipal Park of Muritiba (12°33′44″ S, 41°23′58″ W), in Lençóis; and *S. hatschbachii* was examined at Vaccaro Farm (13°32′01.5″ S, 41°52′17.0″ W) and Pico das Almas (13°32′59.6″ S, 41°55′59.8″ W), in Rio de Contas. All study sites are located within the Chapada Diamantina region. The dominant regional vegetation is campos rupestres, which are ancient, climatically buffered, and nutrient-poor landscapes classified as OCBILs (old, climatically buffered, infertile landscapes) [2].

The Chapada Diamantina exhibits climatic variations driven by altitude, resulting in distinct thermal and precipitation regimes across its slopes. The Municipal Park of Muritiba and the Municipal Natural Park of Pai Inácio fall within a tropical climate regime (Am/Aw), with mean annual temperatures ranging from 22 to 24 °C and annual precipitation between 1100 and 1300 mm, concentrated mainly between November and March. In contrast, sites located in Rio de Contas experience a highland subtropical climate (Cwb), characterized by significantly lower mean annual temperatures (17–19 °C) and slightly reduced annual precipitation, approximately 800 to 1000 mm, with mild summers and dry, cooler winters [68,69].

Due to logistical constraints, the wide geographic distances among species populations, and the absence of flowering in some taxa during the study period, we investigated the floral biology, pollination, and reproductive biology of four species (*S. amoena* Ule, *S. burle-marxii* (L.B.Sm. & Read) Louzada & Wand., *S. hatschbachii* (Leme) Louzada & Wand., and *S. ophiuroides* (Louzada & Wand.) Louzada & Wand.). Floral morphology, pollen morphology and viability, and stigmatic morphology and receptivity were assessed in these four species and four other species (*S. albopicta* (Philcox) Louzada & Wand., *S. heleniceae* (Leme) Louzada & Wand., *S. mucugensis* (Wand. & A.A. Conc.) Louzada & Wand., and *S. ulei* (Louzada & Wand.) Louzada & Wand.), totaling eight species.

### 4.2. Floral Biology and Morphology

We monitored floral life cycle events from bud development to senescence. The timing, sequence, and duration of anthesis were recorded in 10 pre-anthesis floral buds from a single individual for each species, observed between 00:00 and 18:00 over three consecutive days within a two-week period. For morphological analyses, five flowers from different individuals were collected and preserved in 70% ethanol to maintain the floral architecture. Measurements of sepals, petals, and reproductive structures were obtained using a Leica EZ4 D stereomicroscope (Wetzlar, Germany) and digital caliper. Floral traits were assessed in the field and included flower color (recorded qualitatively by direct observation), texture (classified according to the tactile characteristics of floral tissues), odor (evaluated by direct sensory assessment during anthesis), shape (based on floral morphology), corolla aperture size (measured with a digital caliper at the widest point of the corolla opening), and resource availability, which was determined by the presence of nectar and pollen during anthesis.

### 4.3. Stigma Morphology and Receptivity

For stigma and style morphometry, three flowers from five different individuals of each species (n = 15 flowers per species; n = 120 flowers across the eight studied species) were measured using ImageJ 1.53s software. The stigma nomenclature followed Brown and Gilmartin [44,70] and Barfuss et al. [71]. Stigma receptivity was evaluated using two histochemical tests: hydrogen peroxide (3%) and α-naphthyl acetate with Fast Blue B salt and acetone staining. Floral development was based on anthesis (fully opened flowers with exposed reproductive structures). Three flowers from different individuals and populations were collected at this stage, with nine replicates each. In the hydrogen peroxide test, the stigmas were carefully excised to avoid damage and immediately immersed in a 3% hydrogen peroxide solution for three minutes. The formation of air bubbles indicates peroxidase activity and, consequently, stigma receptivity [72]. In the α-naphthyl acetate method, stigmas were immersed in the solution for five minutes and subsequently rinsed with distilled water. A dark brown or black coloration indicates esterase activity, reflecting receptive stigmatic surfaces or papillae [73,74]. For both methods, receptivity was classified following an adaptation of Dafni and Maués [75]: (–) no reaction; (+) weak positive reaction; (++) strong positive reaction; and (+++) very strong positive reaction.

### 4.4. Ovary and Ovules Morphology

For morphological characterization of ovaries and ovules, floral buds and flowers at anthesis were collected and fixed in Karnovsky’s solution [76]. After 48 h of fixation, the material was dehydrated through a graded ethanol series (35–100%) for 20 min at each step and subsequently dried to the critical point (Leica EM CPD 300, Balzers, Germany) using liquid CO_2_. The samples were then mounted on metal stubs and sputter-coated with gold (Leica Microsystems, Vienna, Austria). Digital images were obtained using a scanning electron microscope (SEM; JSM-IT300 LV; JEOL, Tokyo, Japan). Longitudinal and transverse sections of the ovaries were examined to characterize the ovary structure, placentation, and ovule arrangement. Ovule morphology was described based on SEM observations using the standard terminology for angiosperm ovules [77]. Representative images were obtained for qualitative comparisons between the species.

### 4.5. Pollen Morphology and Viability

For morphological characterization of pollen grains, anthers were collected at the pre-anthesis stage and fixed in Karnovsky’s solution [76]. After 48 h of fixation, the material was dehydrated through a graded ethanol series (35–100%) for 20 min at each step and subsequently dried to the critical point (Leica EM CPD 300, Balzers, Germany) using liquid CO_2_. The pollen grains were then mounted on metal stubs and sputter-coated with gold (Leica Microsystems, Vienna, Austria). Digital images were obtained using a scanning electron microscope (SEM; JSM-IT300 LV, JEOL, Tokyo, Japan). Pollen grains were described according to the terminology of Punt et al. [78] and Halbritter et al. [79]. Morphometric data of fresh pollen grains were obtained using weak lactic acetolysis (ACLAC 40) [80] and analyzed under an Olympus BX51 microscope equipped with an Olympus DP175 digital camera (Olympus, Tokyo, Japan). Measurements were performed using ImageJ 1.53s software [81] and included polar and equatorial diameters (in equatorial view), larger and smaller equatorial diameters (in polar view), and exine thickness, based on 25 pollen grains. Pollen shape was determined by the ratio between the polar (P) and equatorial (E) diameters [82].

The pollen grain number was estimated using a Neubauer chamber. Anthers from three flowers taken from different individuals at the pre-anthesis stage were individually placed in 2 mL Eppendorf tubes containing 1 mL lactic acid. The suspension was transferred to a chamber, and the pollen grains were counted using an Olympus BX51 microscope equipped with a digital camera. Pollen viability was assessed using two histochemical tests: Alexander’s stain [83] and fluorescein diacetate (FDA) [84]. Anthers from three flowers of different individuals collected at various floral developmental stages were mounted on slides with a drop of stain, covered with a coverslip, and immediately analyzed. Each slide was scanned under a light microscope (Alexander) or a fluorescence microscope with a UV filter (330–385 nm; FDA). A total of 100 pollen grains per slide were counted, with three replicates per method (n = 300 grains per test). In vitro pollen germination was evaluated using pollen collected from the flowers of three individuals at different developmental stages. Anthers were removed, and pollen grains were evenly distributed in Petri dishes containing 25 mL of two culture media: BM medium [85] and SM medium [86]. The dishes were maintained in a growth chamber at 27 ± 1 °C in the dark for 24 h. Germinated pollen grains were counted, and the lengths of 60 pollen tubes were measured. To enhance the contrast, 0.01% toluidine blue was added prior to imaging. Microphotographs were obtained using a Leica EZ4 W stereomicroscope (Leica Microsystems, Germany), and the measurements were performed using ImageJ 1.53s software. Pollen grains were considered germinated when the pollen tube length was equal to or greater than the diameter of the pollen grain.

### 4.6. Nectar Traits

Nectar was collected from five flowers taken from three individuals using a 10 μL Hamilton microsyringe at anthesis (06:00, 09:00, 12:00, 15:00, and 18:00), with five replicates for each time point. The extracted nectar was placed on a portable refractometer to determine the concentration of total soluble solids (°Brix) in the nectar. The total sugar content per flower was estimated by converting these values to mg of sugar per μL, as described by Galetto and Bernadello [87].

### 4.7. Floral Visitors

Direct focal observations of floral visitors of *S. amoena*, *S. burle-marxii*, *S. hatschbachii*, and *S. ophiuroides* were conducted during anthesis on alternate days and at different times, covering the entire flowering period and totaling over 250 h of observation between 2023 and 2025 (for a complete description, please refer to the Supplementary Materials). Records of floral visitors were obtained using a Canon EOS Rebel T6 (Tokyo, Japan) digital camera equipped with an RF 75–300 mm f/4–5.6 lens, along with field notes on visitor behavior, frequency, and timing of resource use. Visitors were identified based on the specialized literature and consultation with taxonomic experts.

### 4.8. Breeding System

To infer the reproductive system, the following treatments and a control were performed: agamospermy, xenogamy (manual cross-pollination using pollen from a different individual), manual self-pollination (through geitonogamy), spontaneous self-pollination, and control pollination. Flowers assigned to all treatments, except the natural pollination control, were bagged before anthesis to prevent visits from floral visitors. In the spontaneous self-pollination treatment, flowers were bagged without any manipulation. Fruit set was evaluated 15 and 30 days after pollination. For each treatment, 20 to 40 flowers were collected from 10 plants at the flower bud stage (balloon stage), except for the control pollination treatment. All treatments, except for the control and spontaneous self-pollination, included emasculation during pre-anthesis to prevent autonomous self-pollination. Manual pollen deposition on the stigma surface was performed during the first few hours after flower opening using tweezers. The autogamy index (AI) was calculated as the ratio of fruit set in spontaneously self-pollinated flowers to fruit set in cross-pollinated flowers. Species were considered autogamous or partially autogamous when AI > 0.30 [88,89]. The self-incompatibility index (ISI) was calculated as the ratio of fruit set from manually self-pollinated flowers to that from naturally cross-pollinated flowers. Species were considered self-compatible when ISI > 0.30 and self-incompatible when ISI < 0.30 [88,89].

The Reproductive Efficiency Index (RE) was calculated as the ratio of fruit set under natural pollination to fruit set following supplemental cross-pollination (xenogamy), following Zapata and Arroyo [90]. This index provides an estimate of pollen limitation, with values close to 1 indicating little or no pollen limitation and lower values indicating greater pollen limitation.

### 4.9. Statistical Analysis

Pollen viability and in vitro pollen germination were evaluated to assess the pollen quality and reproductive performance. Germination percentages were arcsine transformed (√x/100) before analysis. The data were analyzed using analysis of variance (ANOVA). Differences between the culture media were evaluated using the F-test (*p* < 0.01). Nectar variables (volume, sugar concentration, and total sugar content) were analyzed across sampling times using Kruskal–Wallis tests, followed by Dunn’s multiple comparison tests when significant differences were detected. For the reproductive experiments, differences in fruit set among the pollination treatments were assessed using the Kruskal–Wallis test, followed by Dunn’s post hoc test for pairwise comparisons. The reproductive effectiveness (RE) index was calculated as the ratio of fruit set under natural and supplemental cross-pollination. All statistical analyses were performed using R version 4.5.2 [91], adopting a significance level of *p* < 0.05, unless otherwise indicated.

## 5. Conclusions

This study provides the first detailed assessment of the reproductive biology of four *Sincoraea* species and integrates these findings with morpho-functional analyses of eight species. The results indicate that the studied species predominantly exhibit allogamous breeding systems and depend strongly on hummingbird pollination for reproductive success in the *campos rupestres*. Floral traits such as tubular corollas, diurnal anthesis, stigma receptivity patterns, and nectar production were probably selected by hummingbird and may explain the observed plant–floral visitor interactions.

Most species were self-incompatible, highlighting the importance of cross-pollination for reproductive success, whereas *S. burle-marxii* exhibited partial self-compatibility, indicating variation in breeding systems among the evaluated species.

Results indicated the absence of pollen limitation in the sampled populations, indicating that pollinator services were sufficient to maximize reproductive success, reinforcing the notion of efficient and stable pollination systems within the evaluated communities.

Overall, self-incompatibility may increase the vulnerability of *Sincoraea* species because of their reliance on cross-pollinations. Given the increasing threats to *campos rupestres*, conserving plant–floral visitor interactions, floral visitor communities, and habitat connectivity is important for maintaining the reproductive function and long-term persistence of these species. Future studies incorporating a broader representation of *Sincoraea* and integrating reproductive biology, pollination ecology, population genetics, and phylogenetic approaches will help determine whether the patterns identified here are representative of the genus and provide a stronger basis for conservation and management strategies in these biodiversity hotspots.

## Figures and Tables

**Table 1:** Flower dimensions of *Sincoraea* based on measurements of five flowers per species (n = 5).

Species	Flower Length (cm)		Flower Width (cm)	
	Mean	SD	Mean	SD
*S. amoena*	1.71	0.12	0.49	0.07
*S. burle-marxii*	3.41	0.14	0.48	0.05
*S. hatschbachii*	1.76	0.08	0.38	0.04
*S. ophiuroides*	1.53	0.01	0.54	0.03

**Table 2:** Percentage of fruits with seeds and number of seeds produced under different reproductive systems in four species of *Sincoraea*. Values for fruit set are presented as percentages, with the number of fruits formed relative to the number of flowers tested shown in parentheses (n = 5–40 flowers per treatment, depending on species). Seed production is expressed as mean ± SD number of seeds per fruit. AI = autofertility index; ISI = self-incompatibility index; RE = reproductive efficacy.

**Species**	**Control Pollination**	**Manual Cross-Pollination (Xenogamy)**	**Spontaneous Self-Pollination**	**Artificial Self-Pollination**	**Agamospermy**
	Number of Fruits *				
*S. amoena*	100% (20/20)	100% (20/20)	15% (3/20)	10% (2/20)	0% (0/5)
*S. burle-marxii*	100% (40/40)	100% (25/25)	30% (6/20)	40% (8/20)	0% (0/10)
*S. hatschbachii*	100% (20/20)	100% (20/20)	11% (2/18)	10% (2/20)	0% (0/5)
*S. ophiuroides*	100% (25/25)	100% (20/20)	25% (5/20)	10% (2/20)	0% (0/8)
	Number of Seeds per Fruit **				
*S. amoena*	62.33 ± 6.15	47.08 ± 6.73	6.50 ± 5.54	11.67 ± 7.06	0.00
*S. burle-marxii*	111.17 ± 8.27	84.33 ± 14.46	15.83 ± 10.19	23.33 ± 14.07	0.00
*S. hatschbachii*	89.17 ± 4.76	63.58 ± 8.90	2.08 ± 3.63	3.42 ± 3.50	0.00
*S. ophiuroides*	87.75 ± 6.28	69.08 ± 10.09	5.42 ± 4.42	3.33 ± 2.74	0.00
**Species**	**Indexes**				
	**AI**	**ISI**	**RE**	**Breeding system**	
*S. amoena*	0.10	0.15	1.00	SI—self-incompatible	
*S. burle-marxii*	0.40	0.30	1.00	PSC—partially self-compatible	
*S. hatschbachii*	0.10	0.11	1.00	SI—self-incompatible	
*S. ophiuroides*	0.10	0.25	1.00	SI—self-incompatible	

* Percentage of fruit set (fruits with seeds/pollinated flowers). ** Mean ± standard deviation of seeds obtained per flower. AI = autogamy index (artificial self-pollination/cross-pollination).

## Data Availability

The original contributions presented in this study are included in the article/Supplementary Materials. Further inquiries can be directed to the corresponding author.

## Supplementary Materials

The following supporting information can be downloaded at: https://www.mdpi.com/article/10.3390/plants15142184/s1, File S1: Pollen_Stigma_and_Breeding_system: Table S1. Morphometric characteristics of the stigma and style of eight species of *Sincoraea*. (CM) Length. (Ø) Diameter. Table S2. Stigma receptivity of eight species of *Sincoraea*, evaluated at anthesis by α-naphthyl acetate + fast blue B salt. Table S3. Morphometric characters of the ovary and ovule of four species of *Sincoraea.* Table S4. Morphometric characteristics of pollen grains from eight species of *Sincoraea.* Table S5. Morphological characteristics of pollen grains from eight species of *Sincoraea*. Table S6. Percentage of pollen grain viability in eight species of *Sincoraea* in two histochemical tests (Alexander and Fluorescein diacetate) and during anthesis. Table S7. Quantification of pollen grains and ovules and pollen/ovule ratio of eight species of *Sincoraea*. File S2: Sampling_FloralVisitors.
