# Supplementary material for: Floral Traits and Breeding Systems in Sincoraea (Bromeliaceae), an Endemic Genus of Brazilian Rupestrian Grasslands
Source: Plants (Basel). 2026 Jul 16;15(14):2184. doi: 10.3390/plants15142184 (PMC13414785; doi:10.3390/plants15142184)
Supplement: Supplementary file 1 [file plants-15-02184-s001.zip › File_S1_Pollen_Stigma_and_Breeding_system.pdf]

SUPPLEMENTARY MATERIAL

Floral Traits and Breeding Systems in *Sincoraea* (Bromeliaceae), an Endemic Genus of Brazilian Rupestrian Grasslands

Adelly Cardoso de Araujo Fagundes, Jamerson Souza da Costa, Alexsandro Bezerra-Silva, Maria Thereza Dantas Gomes, Mônica Lanzoni Rossi, Everton Hilo de Souza, Isabel Cristina Sobreira Machado, Ligia Silveira Funch & José Alves de Siqueira Filho

S1 — TABLES

Table S1. Morphometric characteristics of the stigma and style of eight species of *Sincoraea*. (CM) Length. (Ø) Diameter.

Species	Stigma (µm)		Style (µm)	
	Length	Width	Length	Width
S. albopicta	786.25 ± 52.32	382.87 ± 23.32	790.12 ± 47.35	359.32 ± 23.15
S. amoena	23.21 ± 42.18	372.99 ± 18.25	482.03 ± 36.18	351.18 ± 19.15
S. burle-marxii	623.52 ± 33.18	396.83 ± 23.12	678.24 ± 23.98	361.13 ± 25.64
S. hatschbachii	412.35 ± 29.56	355.15 ± 28.56	421.64 ± 30.98	305.18 ± 21.11
S. heleniceae	395.18 ± 36.51	315.18 ± 18.25	402.25 ± 23.98	306.33 ± 23.65
S. mucugensis	728.15 ± 23.57	278.74 ± 31.05	752.22 ± 21.04	228.18 ± 28.15
S. ophiuroides	633.18 ± 47.45	372.15 ± 26.45	647.55 ± 34.12	321.87 ± 40.05
S. ulei	428.53 ± 31.28	289.68 ± 11.18	437.91 ± 30.44	295.75 ± 26.25

Species	Pappillae		Type	Color
	Length	Width		
S. albopicta	40.29 ± 8.25	13.21 ± 0.28	Simple-erect	White
S. amoena	9.12 ± 1.12	6.23 ± 0.38	Simple-erect	White
S. burle-marxii	18.65 ± 2.89	10.18 ± 0.15	Simple-erect	Yellowish-green
S. hatschbachii	7.21 ± 1.05	7.23 ± 052	Simple-erect	Yellowish-green
S. heleniceae	15.15± 3.21	6.18 ± 0.19	Simple-erect	White
S. mucugensis	46.93 ± 4.37	10.23 ± 0.11	Simple-erect	White
S. ophiuroides	7.23 ± 2.01	5.23 ± 0.41	Simple-erect	White
S. ulei	22.16 ± 7.18	12.13 ± 0.23	Simple-erect	White

[1] According to Brown and Gilmartin (1984; 1989) and Barfuss et al. (2016).

Table S2. Stigma receptivity of eight species of *Sincoraea*, evaluated at anthesis by α-naphthyl acetate + fast blue B salt.

**Species**	**α-naftil-acetate + fast blue B salt**
S. albopicta	+++
S. amoena	+++
S. burle-marxii	+++
S. hatschbachii	+++
S. heleniceae	+++
S. mucugensis	+++
S. ophiuroides	+++
S. ulei	+++

Table S3. Morphometric characters of the ovary and ovule of four species of *Sincoraea*.

**Ovary/Ovule Traits**	*S. amoena*	*S. burle-marxii*	*S. hatschbachii*	*S. ophiuroides*
OVL	2.526,21 ± 232,18	6.932,15 ± 374,23	3.978,83 ± 263,14	4.155,90 ± 301,45
OVD	2.786,29 ± 108,25	4.507,80 ± 221,12	3.568,55 ± 204,32	4.340,76 ± 120,44
LOL	403,43 ± 10,11	314,01 ± 13,14	391,93 ± 9,85	3.095,77 ± 78,95
LTW	175,40 ± 10,32	207,82 ± 6,25	262,90 ± 5,33	861,91 ± 25,13
OUL	1.707,66 ± 89,32	3.001,11 ± 102,12	2.566,53 ± 86,54	382,11 ± 7,58
OUD	698,59 ± 23,12	683,74 ± 28,96	766,13 ± 30,12	182,47 ± 9,23
FUNL	428,20 ± 18,13	430,29 ± 12,15	449,39 ± 16,10	396,42 ± 11,82
FUND	103,63 ± 11,13	108,63 ± 8,25	92,14 ± 6,52	78,71 ± 10,12
MPW	54,23 ± 6,31	57,01 ± 8,21	75,00 ± 9,13	56,17 ± 4,25
PLA	Axilary	Axilary	Axilary	Axilary
OVP	Median	Median	Apical	Apical
OVT	Anatropous	Anatropous	Anatropous	Anatropous
MPF	Circular	Circular	Circular	Circular
OVF	Ovoid	Ovoid	Ovoid	Ovoid
CHA	Absent	Absent	Absent	Absent

OVL = ovary length; OVD = ovary diameter; LOL = locule length; LTW = locule transverse width; OUL = ovule length; OUD = ovule diameter; FUNL = funiculus length; FUND = funiculus diameter; MPW = width of the micropylar aperture; PLA = placentation type; OVP = position of the ovules along the placenta; OVT = ovule type; MPF = morphology of the micropylar aperture; OVF = ovule shape; CHA = chalazal appendage.

Table S4. Morphometric characteristics of pollen grains from eight species of *Sincoraea.*

Species	Equatorial view[1]		Polar view[1]		P/E[2]
	Polar diameter (P) (μm)	Equatorial diameter (E) (μm)	Major equatorial (μm)	Minor equatorial (μm)	
S. albopicta	25.30 ± 0.88	33.82 ± 0.74	25.95 ± 0.58	23.96 ± 0.83	0.75
S. amoena	19.81 ± 0.12	32.93 ± 0.18	20.37 ± 0.75	18.62 ± 0.55	0.60
S. burle-marxii	23.79 ± 0.75	34.18 ± 0.68	22.95 ± 0.82	19.93 ± 0.68	0.70
S. hatschbachii	22.04 ± 0.28	30.06 ± 0.26	21.59 ± 0.49	20.02 ± 0.45	0.73
S. heleniceae	21.15 ± 0.57	33.21 ± 0.72	22.09 ± 0.52	20.85 ± 0.76	0.64
S. mucugensis	20.95 ± 0.74	31.12 ± 0.59	20.12 ± 0.72	18.95 ± 0.69	0.67
S. ophiuroides	22.75 ± 0.63	32.18 ± 0.75	23.05 ± 0.32	21.95 ± 0.71	0.71
S. ulei	25.28 ± 0.19	35.13 ± 0.46	26.11 ± 0.33	24.79 ± 0.16	0.72

Species	Thickness[1]		Diameter[1]	Number of pollen grains[3]
	Exine (μm)	Tectum (μm)	Lumen (μm)	
S. albopicta	1.42 ± 0.18	0.31 ± 0.09	0.97 ± 0.54	55,644
S. amoena	1.21 ± 0.20	0.18 ± 0.05	1.14 ± 0.25	41,844
S. burle-marxii	1.52 ± 0.23	0.29 ± 0.03	1.29 ± 0.32	77,889
S. hatschbachii	1.78 ± 0.11	0.48 ± 0.05	1.03 ± 0.25	66,944
S. heleniceae	1.43 ± 0.14	0.31 ± 0.03	0.95 ± 0.33	55,667
S. mucugensis	1.18 ± 0.16	0.12 ± 0.07	0.89 ± 0.41	36,944
S. ophiuroides	1.47 ± 0.20	0.29 ± 0.09	1.23 ± 0.33	62,278
S. ulei	1.52 ± 0.23	0.31 ± 0.10	2.50 ± 0.33	54,555

[1]Pollen grains were subjected to ACLAC 40 according to Raynal and Raynal (1979). The results correspond to the mean of 25 replicates ± standard deviation; [2]According to the method described by Punt et al. (2007) and Halbritter et al. (2018). [3]According to the methodology of Kearns and Inouye (1993).

Table S5. Morphological characteristics of pollen grains from eight species of *Sincoraea*.

**Species**	**Shape[1]**	**Polarity[1]**	**Amb[1]**	**Symmetry [1]**
S. albopicta	Oblate	Heteropolar	Circular	Bilateral
S. amoena	Oblate	Heteropolar	Circular	Bilateral
S. burle-marxii	Oblate	Heteropolar	Circular	Bilateral
S. hatschbachii	Oblate	Heteropolar	Circular	Bilateral
S. heleniceae	Oblate	Heteropolar	Circular	Bilateral
S. mucugensis	Oblate	Heteropolar	Circular	Bilateral
S. ophiuroides	Oblate	Heteropolar	Circular	Bilateral
S. ulei	Oblate	Heteropolar	Circular	Bilateral

**Species**	**Aperture[1]**	**Exine[1]**	**Ornamentation[1]**
S. albopicta	Sulcate	Semitectate	Microreticulate
S. amoena	Sulcate	Semitectate	Reticulate
S. burle-marxii	Sulcate	Semitectate	Reticulate
S. hatschbachii	Sulcate	Semitectate	Reticulate
S. heleniceae	Sulcate	Semitectate	Microreticulate
S. mucugensis	Sulcate	Semitectate	Microreticulate
S. ophiuroides	Sulcate	Semitectate	Reticulate
S. ulei	Sulcate	Semitectate	Reticulate

[1]According to Punt et al. (2007) and Halbritter et al. (2018).

Table S6. Percentage of pollen grain viability in eight species of *Sincoraea* in two histochemical tests (Alexander and Fluorescein diacetate)and during anthesis.

Species	Alexander
S. albopicta	93.08 ± 3.82 aA
S. amoena	85.83 ± 2.92 bA
S. burle-marxii	91.25 ± 3.31 aA
S. hatschbachii	82.33 ± 2.50 bA
S. heleniceae	81.00 ± 3.67 bA
S. mucugensis	80.25 ± 4.03 bA
S. ophiuroides	82.83 ± 2.12 bA
S. ulei	92.83 ± 2.37 aA
CV (%)	5.54
	**Fluorescein diacetate**
S. albopicta	92.42 ± 2.97 aA
S. amoena	83.42 ± 4.74 aA
S. burle-marxii	91.92 ± 3.63 aA
S. hatschbachii	83.92 ± 2.43 aA
S. heleniceae	83.50 ± 1.78 aA
S. mucugensis	85.25 ± 3.11 aA
S. ophiuroides	84.17 ± 2.95 aA
S. ulei	91.42 ± 3.03 aA
CV (%)	6.00

Averages followed by the same lowercase letter in the column and uppercase letter in the row do not differ from each other according to the Scott-Knott and Tukey tests ($p < 0.01$), respectively.

Tabela S7. Quantification of pollen grains and ovules and pollen/ovule ratio of eight species of *Sincoraea*.

Species	Number of Pollen Grains (P)	Number of Ovules (O)	P/O
S. albopicta	55.644	114	489
S. amoena	41.844	74	570
S. burle-marxii	77.889	116	672
S. hatschbachii	66.944	92	727
S. heleniceae	55.667	95	584
S. mucugensis	36.944	72	516
S. ophiuroides	62.278	91	685
S. ulei	54.555	116	472
CV (%)	12.23	5.23	4.32
