# Supplementary material for: Floral Traits and Breeding Systems in Sincoraea (Bromeliaceae), an Endemic Genus of Brazilian Rupestrian Grasslands
Source: Plants (Basel). 2026 Jul 16;15(14):2184. doi: 10.3390/plants15142184 (PMC13414785; doi:10.3390/plants15142184)
Supplement: Supplementary file 1 [file plants-15-02184-s001.zip › File_S2_Sampling_FloralVisitors.pdf]

SUPPLEMENTARY MATERIAL

Floral Traits and Breeding Systems in *Sincoraea* (Bromeliaceae), an Endemic Genus of Brazilian Rupestrian Grasslands

Adelly Cardoso de Araujo Fagundes, Jamerson Souza da Costa, Alexsandro Bezerra-Silva, Maria Thereza Dantas Gomes, Mônica Lanzoni Rossi, Everton Hilo de Souza, Isabel Cristina Sobreira Machado, Ligia Silveira Funch & José Alves de Siqueira Filho

S2 — Sampling dates, observation effort (hours), and geographic coordinates of focal observations of floral visitors in *Sincoraea* species.

Species	Sampling dates	Observation effort (hours)	Longitude	Latitude
Sincoraea amoena	22/01/2023	8	-41.47247593	-12.45716753
Sincoraea amoena	23/01/2023	4	-41.47247593	-12.45716753
Sincoraea amoena	23/01/2023	2	-41.47247593	-12.45716753
Sincoraea amoena	24/01/2023	8	-41.47300598	-12.45795935
Sincoraea amoena	26/01/2023	3	-41.47282956	-12.45743599
Sincoraea amoena	21/01/2024	4	-41.47282956	-12.45743599
Sincoraea amoena	22/01/2024	6	-41.47300598	-12.45795935
Sincoraea amoena	23/01/2024	3	-41.47300598	-12.45795935
Sincoraea amoena	24/01/2024	3	-41.47300598	-12.45795935
Sincoraea amoena	19/01/2026	2	-41.47282956	-12.45743599
Sincoraea amoena	20/01/2026	2	-41.47329452	-12.457992
Sincoraea amoena	20/01/2026	4	-41.47329452	-12.457992
Sincoraea amoena	21/01/2026	3	-41.47329452	-12.457992
Sincoraea burle-marxii	25/01/2023	2	-41.42103462	-12.46122757
Sincoraea burle-marxii	25/01/2023	3	-41.42103462	-12.46122757
Sincoraea burle-marxii	17/01/2024	4	-41.39755323	-12.56228693
Sincoraea burle-marxii	18/01/2024	6	-41.39755323	-12.56228693
Sincoraea burle-marxii	19/01/2024	5	-41.39755323	-12.56228693
Sincoraea burle-marxii	19/01/2024	2	-41.39609165	-12.5614321
Sincoraea burle-marxii	20/01/2024	8	-41.39609165	-12.5614321
Sincoraea burle-marxii	23/01/2026	5	-41.42103462	-12.46122757
Sincoraea burle-marxii	23/01/2026	3	-41.42103462	-12.46122757
Sincoraea burle-marxii	24/01/2026	6	-41.42103462	-12.46122757
Sincoraea burle-marxii	24/01/2026	8	-41.42103462	-12.46122757
Sincoraea burle-marxii	25/01/2026	6	-41.39755323	-12.56228693

Sincoraea hatschbachii	09/12/2025	8	-41.87131845	-13.53385324
Sincoraea hatschbachii	10/12/2025	8	-41.87131845	-13.53385324
Sincoraea hatschbachii	11/12/2025	3	-41.87131845	-13.53385324
Sincoraea hatschbachii	11/12/2025	5	-41.87131845	-13.53385324
Sincoraea hatschbachii	12/12/2025	4	-41.87131845	-13.53385324
Sincoraea hatschbachii	12/12/2025	4	-41.87131845	-13.53385324
Sincoraea hatschbachii	13/12/2025	7	-41.935842	-13.520519
Sincoraea hatschbachii	13/12/2025	2	-41.935842	-13.520519
Sincoraea hatschbachii	14/12/2025	8	-41.935842	-13.520519
Sincoraea hatschbachii	15/12/2025	5	-41.87131845	-13.53385324
Sincoraea hatschbachii	15/12/2025	8	-41.87131845	-13.53385324
Sincoraea ophiuroides	19/01/2023	4	-41.40319187	-12.5612111
Sincoraea ophiuroides	20/01/2023	8	-41.40319187	-12.5612111
Sincoraea ophiuroides	21/01/2023	8	-41.40457867	-12.56013073
Sincoraea ophiuroides	15/01/2024	8	-41.40319187	-12.5612111
Sincoraea ophiuroides	16/01/2024	8	-41.40319187	-12.5612111
Sincoraea ophiuroides	17/01/2024	6	-41.40319187	-12.5612111
Sincoraea ophiuroides	20/02/2024	8	-41.40319187	-12.5612111
Sincoraea ophiuroides	21/02/2024	8	-41.40319187	-12.5612111
Sincoraea ophiuroides	22/02/2024	8	-41.40319187	-12.5612111
Sincoraea ophiuroides	18/02/2025	8	-41.40319187	-12.5612111
Sincoraea ophiuroides	19/02/2022	8	-41.40457867	-12.56013073
